# Albendazole versus Praziquantel in the Treatment of Neurocysticercosis: A Meta-analysis of Comparative Trials

**DOI:** 10.1371/journal.pntd.0000194

**Published:** 2008-03-12

**Authors:** Dimitrios K. Matthaiou, Georgios Panos, Eleni S. Adamidi, Matthew E. Falagas

**Affiliations:** 1 Alfa Institute of Biomedical Sciences (AIBS), Athens, Greece; 2 1st IKA Hospital, Athens, Greece; 3 National Technical University of Athens, Athens, Greece; 4 Tufts University School of Medicine, Boston, Massachusetts, United States of America; University of Oklahoma Health Sciences Center, United States of America

## Abstract

**Background:**

Neurocysticercosis, infection of the brain with larvae of *Taenia solium* (pork tapeworm), is one of several forms of human cysticercosis caused by this organism. We investigated the role of albendazole and praziquantel in the treatment of patients with parenchymal neurocysticercosis by performing a meta-analysis of comparative trials of their effectiveness and safety.

**Methods and Principal Findings:**

We performed a search in the PubMed database, Cochrane Database of Controlled Trials, and in references of relevant articles. Six studies were included in the meta-analysis. Albendazole was associated with better control of seizures than praziquantel in the pooled data analysis, when the generic inverse variance method was used to combine the incidence of seizure control in the included trials (patients without seizures/[patients×years at risk]) (156 patients in 4 studies, point effect estimate [incidence rate ratio] = 4.94, 95% confidence interval 2.45–9.98). In addition, albendazole was associated with better effectiveness than praziquantel in the total disappearance of cysts (335 patients in 6 studies, random effects model, OR = 2.30, 95% CI 1.06–5.00). There was no difference between albendazole and praziquantel in reduction of cysts, proportion of patients with adverse events, and development of intracranial hypertension due to the administered therapy.

**Conclusions:**

A critical review of the available data from comparative trials suggests that albendazole is more effective than praziquantel regarding clinically important outcomes in patients with neurocysticercosis. Nevertheless, given the relative scarcity of trials, more comparative interventional studies—especially randomized controlled trials—are required to draw a safe conclusion about the best regimen for the treatment of patients with parenchymal neurocysticercosis.

## Author Summary

Neurocysticercosis is a parasitic disease caused by the pork tapeworm, *Taenia solium*, when the larval form of the parasite lodges in the central nervous system. This disease is most commonly found among members of agricultural societies with poor sanitary conditions and economies based on breeding livestock (especially pigs) with low hygiene standards. It is a disease with long history in humans, and the usual therapeutic intervention was surgery until the development of antiparasitic cysticidal agents, the most common being praziquantel and albendazole. *T. solium* infection can take many different forms in humans, but we concentrated on parenchymal neurocysticercosis with viable cysts. A consensus statement by a panel of experts on the subject supports the use of antiparasitic treatment, but does not indicate either albendazole or praziquantel as the drug of choice for this type of neurocysticercosis, because data from single relevant clinical trials are not conclusive. We conducted a meta-analysis to further evaluate the comparative effectiveness and safety of albendazole and praziquantel for this particular type of neurocysticercosis. The outcomes of our meta-analysis suggest that albendazole is more effective than praziquantel in controlling seizures in affected patients and in leading to the total disappearance of cysts and subsequently cure of patients with neurocysticercosis.

## Introduction

Neurocysticercosis is a parasitic disease caused by the larval form of *Taenia solium*, known as pork tapeworm, when the larvae lodge in the central nervous system (CNS). It happens when human ingests the eggs, acting as the intermediate host in the life cycle of *T. solium*. The eggs hatch in the intestine and the embrya penetrate the intestinal wall and are distributed via the blood, anchoring in the CNS as a larval form of the parasite [1]. With *T. solium* parasitosis, both self-reinfection and infection of household members are common.

Neurocysticercosis is mosst commonly found among members of agricultural societies with poor sanitary conditions and economies based on breeding livestock, especially pigs, with low hygiene standards [2]. However, it has also started to emerge in developed countries, as a result of immigration from endemic to nonendemic areas [3]. Its natural pool lies mainly in Latin America, sub-Saharan Africa, and Southeast Asia, and is an important cause of morbidity among local populations [2].

Neurocysticercosis is divided into four categories depending on the anatomical locus in which the larvae lodge—cerebral or parenchymal, subarachnoid or cisternal, intraventricular, and spinal [1]. The most common clinical sign of neurocysticercosis is epilepsy of any type, which is usually late-onset; this sign is typically found in parenchymal neurocysticercosis. Other common signs are focal neurological deficits, cerebellar or brainstem signs, signs of increased intracranial pressure, meningoencephalitic signs, dementia, or even death [4].

The standard therapeutic intervention was surgery until the development of cysticidal agents, the most common being praziquantel and albendazole [5]. Although there have been many clinical trials testing these drugs, controversy remains about their therapeutic value [5]. The reasons for this dispute include the severity of adverse effects, the actual reduction of cysts, and the subsequent control of seizures. This disagreement seems to have been resolved after the recent publication of a meta-analysis that shows the superiority of these agents compared to placebo [6].

We sought to investigate which of the two agents are preferable in the treatment of neurocysticercosis. Some studies have been published on this issue, although they mostly examine small numbers of patients. Specifically, we investigated the role of albendazole versus praziquantel in the treatment of patients with parenchymal neurocysticercosis by performing a meta-analysis of comparative trials [7] of their effectiveness and safety.

## Methods

### Data sources

The studies for our meta-analysis were obtained from the PubMed database, Cochrane Database of Controlled Trials, and from references of relevant articles. Search terms included “albendazole”, “praziquantel”, “neurocysticercosis”, and “*Taenia solium*”. Although the search was performed without limitation on the language of publications, the evaluable studies were published in English, French, German, and Italian. There was no limitation on the year of publication.

### Study selection

Two independent reviewers (DKM and GP) performed the search and selected the studies that were relevant to the scope of our meta-analysis. Any discrepancy or disagreement between the reviewers was resolved by consensus in meetings involving all authors. A study was considered eligible if (1) it was a prospective trial, (2) it compared albendazole with praziquantel for the treatment of patients with neurocysticercosis, (3) it examined the partial or total disappearance of cysts and/or control of seizures, and (4) if it included patients infected with parasites in their cystic stage without perilesional inflammation. Studies using concomitant drugs such as corticosteroids, analgesics, and anticonvulsive drugs were not excluded.

### Data extraction

The following data were extracted from each study: year of publication, study design, population of the study, therapeutic regimens used, concomitant drugs, number of patients, follow-up period, patients having control of seizures, proportion of cyst reduction, disappearance of cysts, total toxicity, and patients presenting intracranial hypertension as a side effect. A quality review of each randomized controlled trial (RCT) included in our analysis was performed by using the Jadad score, which examines whether there is randomization, blinding, and information on withdrawals in the study, and evaluates the appropriateness of randomization and blinding, if present. One point was awarded for the presence of each of the first 3 criteria, whereas the last 2 criteria could take the values of −1 (inappropriate), 0 (no data), and +1 (appropriate) [8],[9]. Thus, the maximum score for a study was 5, and a score more than 2 points denoted an adequate RCT according to the methodology. The reviewers calculated the score of each RCT independently. Any disagreement was resolved after consensus among all authors.

### Outcomes

The primary outcome was the proportion of patients with controlled seizures. Secondary outcomes were the reduction of cysts in all of the examined patients, the proportion of patients with total disappearance of cysts, the proportion of patients with adverse events related with the administered antihelminthic drugs, and the proportion of patients with intracranial hypertension as a side effect caused by the administered drugs.

### Definitions

A patient was considered as having total control of seizures when there had been no seizures during the follow-up period. A patient was considered as having total disappearance of cysts when this outcome had been achieved after only one course of administered chemotherapy and without any surgical intervention at the follow-up CT scan, performed in a time frame of 3 to 6 months after the end of therapy. The reduction of cysts was defined as the proportion of the number of cysts that had resolved by the follow-up evaluation (numerator), which varied from 3 to 6 months post-therapy, divided by the number of cysts at baseline (denominator). Adverse events included any type of adverse event reported in the included studies.

### Statistical analyses

Statistical analyses were performed using the “Review Manager 4.2” software and the SPSS 15.0 statistical software. The heterogeneity between studies was assessed by using the I^2^ test and χ^2^ test; for the χ^2^ test, p<0.10 was considered statistically significant in the analysis of heterogeneity [10]. Small-study bias was assessed by the funnel plot method [11]. Pooled odds ratios (ORs) and 95% confidence intervals (CIs) for all primary and secondary outcomes were calculated by using both the Mantel-Haenszel [12] fixed effect model and the DerSimonian-Laird random effects model [13]. For all analyses, results from the fixed effect model are presented only when there was no heterogeneity between studies; otherwise, results from the random effects model are presented. For the analyses of proportions of the reduction of cysts, we used a linear regression model in which the percentage of reduction of cysts for each treatment arm in the included studies was the dependent variable, and the administered drug was the independent variable. With this model, a beta (β) coefficient of the independent variable was calculated as well as the 95% confidence interval (CI) of the coefficient. For the analyses of seizure control for which the follow up period varied, we combined the logarithms of the rate ratios across the included trials (patients with outcome/[patients×years at risk]) using the generic inverse variance method.

## Results

### Selection of the trials

**Figure 1:**
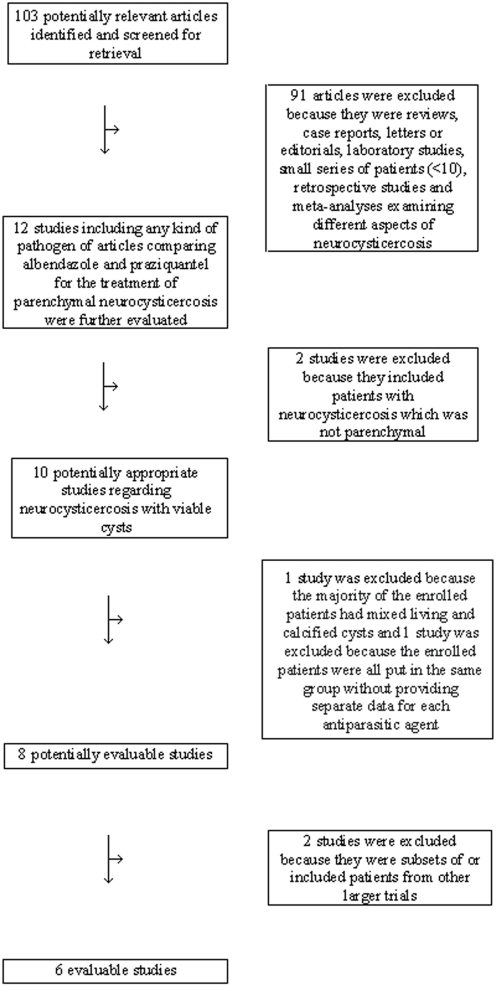
Flow diagram of reviewed articles.

Figure 1 is a flow diagram describing the process of study selection. We identified 103 potentially evaluable papers, 91 of which were excluded because they were reviews, case reports, letters or editorials, laboratory studies, small series of patients, retrospective studies, and meta-analyses that examined a different aspect of neurocysticercosis than the comparison between praziquantel and albendazole. Of the remaining 12 potentially evaluable papers, 2 studies were excluded because they included patients with neurocysticercosis that was not parenchymal, 1 because the majority of the enrolled patients had mixed living and calcified cysts, 1 because the enrolled patients were all put in the same group without providing separate data for each antiparasitic agent, and 2 because they were subsets of other larger trials. Thus, 6 trials were included in our meta-analysis [14]–[19].

### Quality assessment

The assessment of the evaluable studies according to the Jadad score was performed only for the 2 out of 6 studies [15],[19]. The rest of the studies were prospective [14], [16]–[18] but not RCTs. Thus, quality assessment of these trials using Jadad could not be done.

### Administration of study drugs

The studies differed in the administered dosing and duration of therapy for albendazole and praziquantel (Table 1). Most of the researchers administered 15 mg/kg/d of body weight of albendazole, but the duration of therapy varied from 8 days to a month [14]–[16],[18],[19]. In only one study albendazole was administered at a dosage of 20 mg/kg/d for 21 days [17]. We pooled these data, as the administration of albendazole for 7 days is as effective as for longer periods of therapy [20]. There was notable variation in the duration of praziquantel therapy, extending from a single day to 3 weeks. In all of the studies the dosage of praziquantel was 50 mg/kg/d, except one study in which praziquantel was administered at a dosage of 100 mg/kg in 3 divided doses at 2-hour intervals for a single day [14]. We pooled these data, as the administration of praziquantel for a single day is as effective as for longer periods of therapy [21]–[23].

**Table 1:** Main characteristics of the selected trials.

Author-Year	Type of infection-Setting-Country	Jadad score*	1^st^ regimen	2^nd^ regimen	Concomitant therapy	No. of patients (total-ALB-PZQ)	Follow-up
Del Brutto et al 1999 [14]	Prospective study - Parenchymal NCC with viable cysts and without CT evidence of surrounding inflammation - Ecuador	—	**Albendazole** 15 mg/kg/d in two divided doses for 1 week	**Praziquantel** 100 mg/kg in three divided doses at 2 hour intervals for 1 day	AED, corticosteroids, analgesics, antiemetics	20-10-10	CT scans after 3 months, 6–12 months total follow-up
Carpio et al 1995 [15]	Open RCT – Active parenchymal NCC without enhancement with contrast media in CT and with or without calcifications - Ecuador	3	**Albendazole** 15 mg/kg/d for 8 days	**Praziquantel** 50 mg/kg/d for 15 days	AED, corticosteroids	111-57-54	CT scans between 3–6 months and 9–12 months, 2 years total follow-up at 2 month intervals
Medina et al 1993 [16]	Prospective study – Parenchymal NCC without CT evidence of surrounding inflammation - Mexico	—	**Albendazole** 15 mg/kg/d for 8 days	**Praziquantel** 50 mg/kg/d for 8 days	AED	16-11-5	CT scans and/or MRI after 3 months, 10–18 months total follow-up at 3 month intervals
Takayanagui et al 1992 [17]	Prospective study - Parenchymal NCC with non-enhancing cystic lesions - Brazil	—	**Albendazole** 20 mg/kg/d for 21 days	**Praziquantel** 50 mg/kg/d for 21 days	AED, corticosteroids	43-21-22	CT scans after 6 months, CSF analyses after 1 month
Cruz et al 1991 [18]	Prospective study –NCC of any developmental phase of the parasite - Ecuador	—	**Albendazole** 15 mg/kg/d for 30 days	**Praziquantel** 50 mg/kg/d for 15 days	AED, corticosteroids, analgesic, symptomatic medication	100-50-50	CT scans after 3 months, 3 months follow-up for recurrence
Sotelo et al 1990 [19]	RCT - Parenchymal NCC without CT evidence of surrounding inflammation - Mexico	3	**Albendazole** 15 mg/kg/d for 1 month (group III) and 15 mg/kg/d for 8 days (group IV)	**Praziquantel** 50 mg/kg/d for 15 days (group I) and 50 mg/kg/d for 8 days (group II)	AED, corticosteroids, analgesics, antiemetics	114-49-65	CT scans after 3 months

*: The two reviewers were concordant in the assessment of the scores of the included studies.

AED, antiepileptic drugs; ALB, Albendazole; CT, computed tomography; MRI, magnetic resonance imaging.NCC, neurocysticercosis; PZQ, Praziquantel; RCT, randomized controlled trial.

### Control of seizures

**Figure 2:**
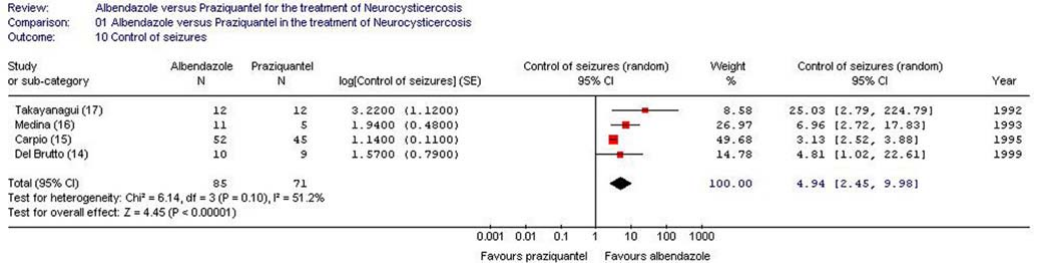
Odds ratios of control seizures in patients with neurocysticercosis treated with albendazole or praziquantel. Vertical line = “no difference” point between the two regimens. Square = odds ratio; the size of each square denotes the proportion of information given by each trial. Diamond = pooled odds ratio for all trials. Horizontal lines = 95% CI.

Data on the complete control of seizures in patients with neurocysticercosis treated with albendazole or praziquantel were reported in 4 out of 6 studies (Table 2) [14]–[17]. One study reported a statistically significant effects in favor of albendazole, as reported in the crude data provided in the study [17]. To overcome the variation in the follow-up periods, we used the generic inverse variance method to combine the incidence of seizure control (patients without seizures/[patients×years at risk]) of the included trials (Table 2). Albendazole was associated with better control of seizures in comparison with praziquantel in the pooled data analysis (156 patients, random effects model [I^2^ = 51.2%], point effect estimate [incidence rate ratio] = 4.94 [seizure-free persons/person-years], 95% CI 2.45–9.98, Figure 2).

**Table 2:** Clinical outcomes including adverse events for patients with neurocysticercosis treated with albendazole versus praziquantel.

Author-Year	Control of seizures		Control of seizures (patients without seizures/[patients×years at risk])		Reduction of cysts		Total disappearance of cysts		Mortality		Patients with adverse events		Intracranial hypertension	
	ALB	PZQ	ALB	PZQ	ALB	PZQ	ALB	PZQ	ALB	PZQ	ALB	PZQ	ALB	PZQ
Del Brutto et al 1999 [14]	10/10 (100%)	7/9 (77.8%)	10/5	7/5.5	57/64 (89.1%)	35/59 (59.3%)	5/10 (50%)	3/10 (30%)	0/10 (0%)	0/10 (0%)	3/10 (30%)	6/10 (60%)	0/10 (0%)	2/10 (20%)
Carpio et al 1995 [15]	33/52 (63.5%)	26/45 (57.8%)	33/71	26/64	129/313 (41.2%)	105/253 (41.5%)	16/57 (28.1%)	17/54 (31.5%)	0/57 (0%)	0/54 (0%)	20/57 (35.1%)	22/54 (40.7%)	NR	
Medina et al 1993 [16]	10/11 (90.9%)	3/5 (60%)	10/9	3/5.25	228/254 (89.8%)	95/120 (79.2%)	8/11 (72.7%)	1/5 (20%)	0/11 (0%)	0/5 (0%)	NR		0/11 (0%)	0/5 (0%)
Takayanagui et al 1992 [17]	11/12 (91.7%)	5/12 (41.7%)	11/3.25	5/4.75	89/101 (88.1%)	89/178 (50%)	11/20 (55%)	3/20 (15%)	0/21 (0%)	1/22 (4.5%)	12/21 (57.1%)	19/22 (86.4%)	1/21 (4.8%)	2/22 (9.1%)
Cruz et al 1991 [18]	NR		NR		NR		8/10 (80%)	19/24 (79.2%)	0/50 (0%)	0/50 (0%)	0/50 (0%)	0/50 (0%)	0/50 (0%)	0/50 (0%)
Sotelo et al 1990 [19]	NR		NR		482/568 (84.9%)	361/655 (55.1%)	32/49 (65.3%)	25/65 (38.5%)	0/49 (0%)	0/65 (0%)	42/49 (85.7%)	49/65 (75.4%)	NR	

ALB, Albendazole; NR, not reported; PZQ, Praziquantel.

### Reduction of cysts

Data on the reduction of the total number of cysts from baseline to follow-up are reported in 5 out of 6 studies (Table 2) [14]–[17],[19]. A linear regression model of the proportion of reduction of cysts and the administration of albendazole or praziquantel yielded a beta coefficient (β) = 0.22 (standard error [SE] = 0.113) with 95% CI −0.05 to 0.48. The analysis included a total of 301 patients with 2565 cysts. Hence, there was no statistically significant difference in the proportion of the reduction of cysts between albendazole and praziquantel for the treatment of neurocysticercosis. In addition, in a sensitivity analysis excluding the data reported in the RCT by Sotelo et al [19] which comprised almost one half of the total number of cysts, there was no statistically significant difference in the proportion of the reduction of cysts between albendazole and praziquantel for the treatment of neurocysticercosis (β = 0.15 [SE = 0.18], 95% CI −0.30 to 0.59). The analysis included a total of 187 patients with 1342 cysts.

### Total disappearance of cysts

**Figure 3:**
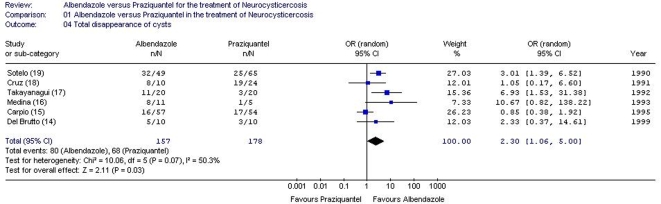
Odds ratios of patients with total disappearance of cysts. Vertical line = “no difference” point between the two regimens. Square = odds ratio; the size of each square denotes the proportion of information given by each trial. Diamond = pooled odds ratio for all trials. Horizontal lines = 95% CI.

Data on the total disappearance of cysts are reported in all 6 studies (Table 2) [14]–[19]. Albendazole was associated with greater efficacy than praziquantel in the total disappearance of cysts (335 patients, random effects model (χ^2^-test p = 0.07, I^2^ = 50.3%), OR = 2.30, 95% CI 1.06–5.00, Figure 3). Since in the study by Cruz et al [18] it is not clear whether the patients with cystic lesions also had lesions involving other stages of the infection, we performed a sensitivity analysis without the aforementioned study, in which albendazole was more effective than praziquantel in inducing the total disappearance of cysts (301 patients, random effects model (χ^2^-test p = 0.05, I^2^ = 58.1%), OR = 2.62, 95% CI 1.09–6.32). We also performed a sensitivity analysis excluding data reported in the RCT by Sotelo et al [19], which included almost one-third of the total number of patients in this meta-analysis and showed statistical significance. There was no difference between the two regimens in inducing the total disappearance of cysts (221 patients, random effects model (χ^2^-test p = 0.08, I^2^ = 52.5%), OR = 2.20, 95% CI 0.79–6.13).

### Mortality

Data about mortality are reported in all 6 studies (Table 2) [14]–[19]. One death was reported in by Takayanagui et al [17] due to increased intracranial pressure. These data were not adequate to allow a meaningful analysis.

### Total adverse events

Data about patients with adverse events are reported in 5 out of 6 studies (Table 2) [14], [15], [17]–[19]. Albendazole and praziquantel did not differ in the proportion of patients with adverse events (388 patients, random effects model [χ^2^-test p = 0.06, I^2^ = 59.9%], OR = 0.67, 95% CI 0.26–1.69).

### Intracranial hypertension

Data on intracranial hypertension developing as a consequence of the regimens administered are reported in 4 studies (Table 2) [14], [16]–[18]. There was no difference in the development of intracranial hypertension due to the administered therapy between albendazole and praziquantel (179 patients, fixed effect model [χ^2^-test p value = 0.58, I^2^ = 0%], OR = 0.31, 95% CI 0.05–2.09).

## Discussion

Neurocysticercosis is an endemic disease in many developing countries, and it may expand to the developed world, mainly as a result of immigration. Estimations report around 50 million new cases worldwide [24]. To our knowledge, until now the guidelines for the treatment of cysticercosis are the result of a consensus by a panel of experts in the subject [25]. Specifically, for viable parenchymal cysts the recommendations are based on evidence obtained from multiple case series with or without intervention, including dramatic results in uncontrolled experiments (level II-3 recommendation, which is considered a weak category of evidence), and on opinions of respected authorities, based on clinical experience, descriptive studies, and case reports or reports of expert committees (level III recommendation). Although these recommendations support the use of antiparasitic treatment, they do not point to either albendazole nor praziquantel as the drug of choice for this type of neurocysticercosis.

In a recent meta-analysis performed by Del Brutto et al. [6] it was suggested that, compared to placebo, cysticidal drug therapy results in better resolution of colloidal and vesicular cysticerci, lower risk for recurrence of seizures in patients with colloidal cysticerci, and a reduction in the rate of generalized seizures in patients with vesicular cysticerci. However, there has not yet been a meta-analysis comparing the effectiveness and safety of albendazole and praziquantel in patients with neurocysticercosis.

The outcomes in our meta-analysis suggest that albendazole is more effective than praziquantel in controlling seizures in the affected patients and in leading to the total disappearance of cysts and, subsequently, the cure of patients with neurocysticercosis. However, in the sensitivity analysis of the total disappearance of cysts, excluding the study by Sotelo et al [19], no significant difference was found between the drugs, although the odds ratio was rather similar to the analysis that included the study by Sotelo et al. [19]. This loss of statistical significance can be explained by the loss of power in the sensitivity analysis due to exclusion of the aforementioned study. Regarding other outcomes, there have been no statistically significant differences between albendazole and praziquantel in reduction of total number of cysts, mortality, total adverse events, and development of intracranial hypertension due to the administered therapeutic agents. Control of seizures and total disappearance of cysts were chosen as outcomes in our meta-analysis, because they are easily defined and quantitatively measured. In addition, new-onset seizures are among the most common symptoms that lead patients to seek medical care, and their resolution is one of the major goals of therapy.

In the analyses of outcomes we did not perform sensitivity analyses that excluded the study by Medina et al [16], in which patients did not receive corticosteroids. Since it is the only study with this characteristic, one may suggest that it could cause bias. It might be speculated that the absence of corticosteroids could interfere with the kinetics of the administered antihelminthics, and cause an increase in the rate of the adverse events. However, all the outcomes included in this study did not differ from the results of the other trials; adverse events are not reported in this study.

The reduced effectiveness of praziquantel could be explained by the interaction between praziquantel and corticosteroids, which results in decreased serum concentration of praziquantel [26]. Also, praziquantel interacts with anti-epileptic drugs [27],[28], thus altering its bioavailability. In contrast, corticosteroids interact with albendazole by decreasing the rate of elimination of albendazole sulfoxide, which is the active metabolite of albendazole, thus increasing serum concentrations of albendazole sulfoxide [29],[30].

Often, the first few days after the administration of antiparasitic agents to patients with neurocysticercosis there is a recrudescence of neurological symptoms, most importantly decompensation of intracranial pressure and the onset of seizures or worsening of pre-existing ones, owing to peri-lesional inflammation due to degeneration of the parasite; this condition can be life-threatening. The severity of inflammation is proportional to the parasitic burden, resulting in more severe manifestations in individuals with greater cyst loads [31]. A common approach to ameliorating this problem is the concomitant administration of corticosteroids to reduce edema, the inflammatory response, and intracranial hypertension [32]. Special attention should be paid to patients with high cyst loads, to whom the administered antiparasitic treatment causes an abrupt degeneration of cysts that may lead to severe inflammation and seizures [5]. In such cases corticosteroids should be administered before the antiparasitic agents. The single death reported in the study by Takayanagui et al [17] (the only death among patients of all trials included in this meta-analysis) was the result of increased intracranial pressure, which, however, pre-existed at the beginning of the trial. In 5 out of 6 studies included in our meta-analysis, corticosteroids were administered to patients [14], [15], [17]–[19]. Only in the study by Medina et al [16] were corticosteroids not administered; adverse events were not reported in this study.

It is believed by several experts that many cysts degenerate spontaneously over time, which may lead to the conclusion that the results of the evaluable studies may be biased [33]. Since it is not clear up to what extent this opinion is true, we analyzed studies that included patients with cystic lesions without perilesional enhancements or other evidence of surrounding inflammation, as evidence of a possible degenerative process, to rule out such a possibility. Antihelminthic drugs are effective against viable cysts, but not on remnants, granulomas, and calcifications of dead cysts. Thus, both outcomes we chose to study—the total disappearance of cysts and reduction of cysts—are useful indicators of the effectiveness of the administered therapy, because they estimate the effectiveness of the administered agents for lesions on which the agents are active.

There are some limitations in our meta-analysis that should be considered. First, one may claim that the number of the studies and the number of patients are too small to allow a definitive conclusion regarding the results of the compared therapies. This small sample size is important because it leads to large confidence intervals. In addition, publication bias cannot be appropriately assessed in a small set of studies. Also, among the studies selected there are only 2 RCTs [15],[19] in a total of 6 comparative trials, which prevents us from applying the usually applied methodology in obtaining an overall quality assessment of the included studies [8].

Second, there are discrepancies in the administered dosage and duration of therapy with the 2 antiparasitic agents used. Although there have been several studies aiming to establish an optimal dosage and duration of therapy, these important therapeutic parameters have not been standardized yet. We pooled all of the available data, since the dosage and the duration of therapy used in the trials included in this meta-analysis are generally accepted alternatives by the medical community.

Furthermore, there were differences in the length of follow-up for the control of seizures between the studies that varied from 6 to 24 months. This fact may give rise to methodological issues regarding the validity of combining these studies without considering the duration of follow-up. Thus, we performed an analysis using the generic inverse variance method combining the incidence of seizure control in the included trials (patients without seizures/[patients×years at risk]), in which the effect of different follow-up time is included. However, it should be noted that the caveat in this methodology is the assumption that the risk for seizures is constant, which is not proven. Despite the aforementioned limitations, the contribution of the meta-analysis in the literature sheds light in the subject given the scarcity of data.

In summary, neurocysticercosis is a disease with a long history in humans and with many different stages. We concentrated on parenchymal neurocysticercosis with viable cysts. The recommendations suggest the administration of antiparasitic treatment with concomitant use of steroids. This meta-analysis sought to provide more accurate estimates of the comparative effectiveness and safety of albendazole and praziquantel for this common parasitic infection. Nevertheless, more studies, especially randomized controlled trials, with homogeneous regimens and long follow-up periods, are required to draw a clear conclusion about the best regimen for the treatment of patients with parenchymal neurocysticercosis.

## Supporting Information

QUOROM Checklist (0.30 MB DOC)
