# Supplementary figures and images for: Albendazole versus Praziquantel in the Treatment of Neurocysticercosis: A Meta-analysis of Comparative Trials
Source: PLoS Negl Trop Dis. 2008 Mar 12;2(3):e194. doi: 10.1371/journal.pntd.0000194 (PMC2265431; doi:10.1371/journal.pntd.0000194)

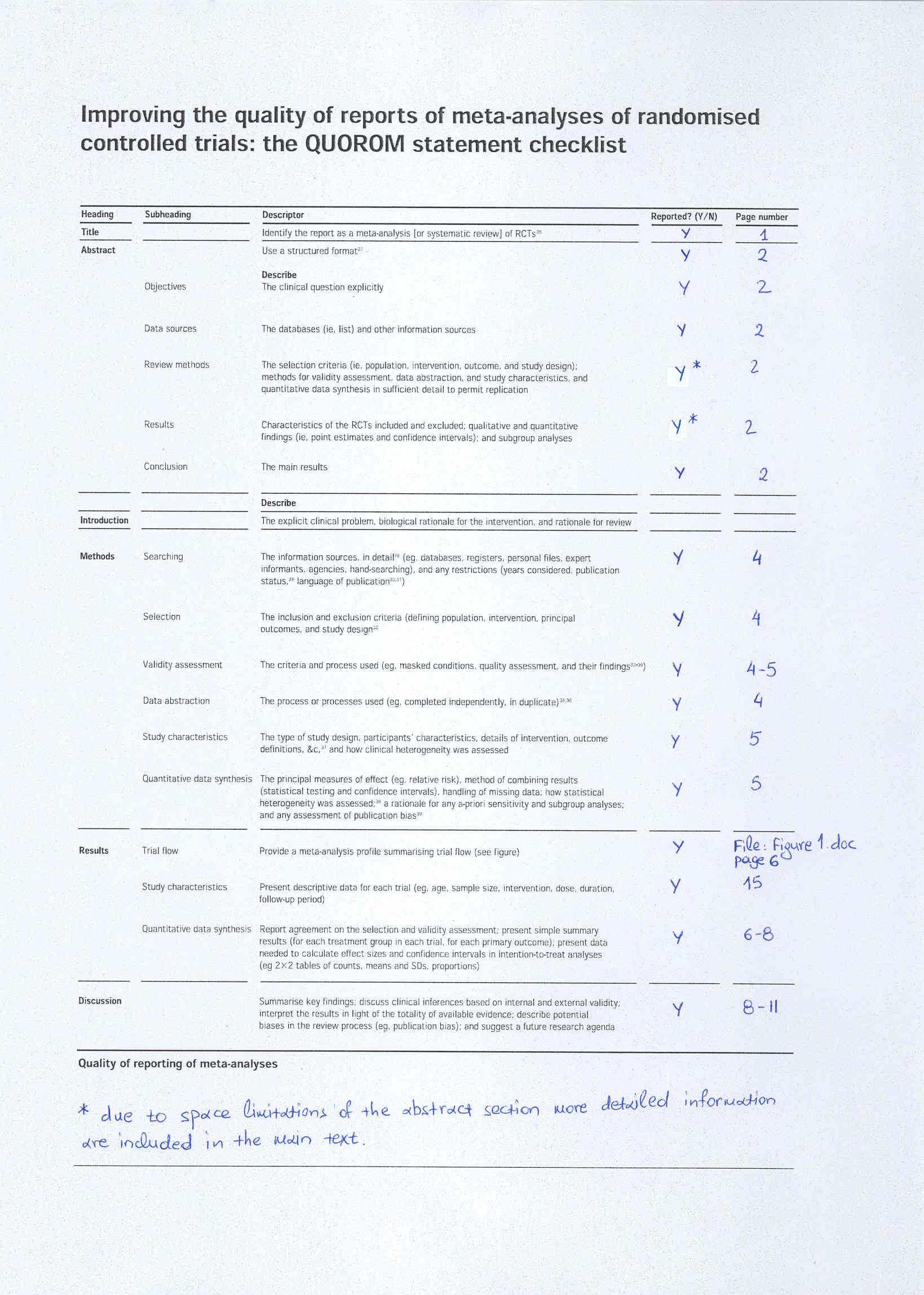

Supplement: QUOROM Checklist — (0.30 MB DOC) [file pntd.0000194.s001.doc]
